# Integrative Analysis of Neuregulin Family Members-Related Tumor Microenvironment for Predicting the Prognosis in Gliomas

**DOI:** 10.3389/fimmu.2021.682415

**Published:** 2021-05-13

**Authors:** Wei-jiang Zhao, Guan-yong Ou, Wen-wen Lin

**Affiliations:** ^1^ Cell Biology Department, Wuxi School of Medicine, Jiangnan University, Wuxi, China; ^2^ Center for Neuroscience, Shantou University Medical College, Shantou, China

**Keywords:** neuregulin family, gliomas, tumor microenvironment, m^6^A modification, GSVA, prognosis

## Abstract

Gliomas, including brain lower grade glioma (LGG) and glioblastoma multiforme (GBM), are the most common primary brain tumors in the central nervous system. Neuregulin (NRG) family proteins belong to the epidermal growth factor (EGF) family of extracellular ligands and they play an essential role in both the central and peripheral nervous systems. However, roles of NRGs in gliomas, especially their effects on prognosis, still remain to be elucidated. In this study, we obtained raw counts of RNA-sequencing data and corresponding clinical information from 510 LGG and 153 GBM samples from The Cancer Genome Atlas (TCGA) database. We analyzed the association of NRG1-4 expression levels with tumor immune microenvironment in LGG and GBM. GSVA (Gene Set Variation Analysis) was performed to determine the prognostic difference of NRGs gene set between LGG and GBM. ROC (receiver operating characteristic) curve and the nomogram model were constructed to estimate the prognostic value of NRGs in LGG and GBM. The results demonstrated that NRG1-4 were differentially expressed in LGG and GBM in comparison to normal tissue. Immune score analysis revealed that NRG1-4 were significantly related to the tumor immune microenvironment and remarkably correlated with immune cell infiltration. The investigation of roles of m^6^A (N6-methyladenosine, m^6^A)-related genes in gliomas revealed that NRGs were prominently involved in m^6^A RNA modification. GSVA score showed that NRG family members are more associated with prognosis in LGG compared with GBM. Prognostic analysis showed that NRG3 and NRG1 can serve as potential independent biomarkers in LGG and GBM, respectively. Moreover, GDSC drug sensitivity analysis revealed that NRG1 was more correlated with drug response compared with other NRG subtypes. Based on these public databases, we preliminarily identified the relationship between NRG family members and tumor immune microenvironment, and the prognostic value of NRGs in gliomas. In conclusion, our study provides comprehensive roles of NRG family members in gliomas, supporting modulation of NRG signaling in the management of glioma.

## Introduction

Gliomas are the most prevalent type of brain tumor derived from brain glial cells, and they have caused considerable morbidity and mortality (1). World Health Organization (WHO) classification system classifies gliomas from grade I to grade IV, in which Grades I and II gliomas are referred to as low grade gliomas (LGG), while grades III and IV gliomas are referred to as high grade gliomas (HGG) (2). LGGs arise from neuroepithelial tissue (3) and account for 10-15% of primary brain tumors (1), 70% of which can inevitably progress to grade IV glioblastoma (GBM) (4). Currently, treatments against glioma are limited and prognosis upon diagnosis tends to be very poor (5). Although surgical resection, chemotherapy and radiation therapy have been considered standard treatments for glioma clinically (6), none of these can cure glioma alone (1, 7). Therefore, a better understanding of the molecular mechanisms underlying the progression of glioma is essential for the development of new treatments that can ultimately improve the prognosis of patients with glioma.

Tumor microenvironment (TME) refers to the environment where the tumor originates, which is highly involved in cancer development (8). TME includes extracellular matrix and various non-transformed cells such as fibroblasts, immune infiltrates, and vascular network recruited from tissues (8). Through providing matrices, cytokines, growth factors and other factors, the TME plays a vital role in tumor development, invasion, metastasis and resistance to therapy, thus influencing the tumor fate (9). Neuregulin (NRG) family members, one of the largest subclasses of the epidermal growth factor family (10) widely expressed in various tissues (11) such as brain (12), heart (13), and breast (14), have long been considered as important molecules in regulating tumor progression (15, 16). In the meantime, previous studies indicated that NGRs play important roles in the initiation and development of human tumors including gliomas (17), gastric cancer (18), Schwannoma (19), colon cancer (20), breast cancer (21) and prostate cancer (22) by regulating cancer cell migration and TME, emerging as therapeutic targets in developing novel strategies against cancers. NRG family members, especially NRG1 and NRG3, have been proved to play important roles in brain development, including neural plasticity (23), differentiation (24), and Schwann cell migration (25). Thus, they may have great potential in the treatment of brain gliomas. Zhao et al. (26) found that NRG1 can regulate the expression of cell adhesion molecule L1 in glioma cells, and may promote malignancy by upregulating the L1 expression in glioblastoma cells. And Lin et al. (27) found that NRG1 may contribute to malignancy by upregulating cell adhesion molecular L1-like protein (CHL1) expression levels in glioma/glioblastoma cells. Patricia et al. (28) found that ErbB receptor activation by NRG1 enhances cell motility that involves the activation of focal adhesion kinase, suggesting that NRG1 plays a crucial modulatory role in glioma cell invasion. Furthermore, NRG1 can enhance survival of human astrocytic glioma cells through autocrine/paracrine pathways under growth restriction (29).

In contrast to these reports, the correlation between prognostic roles of distinct NRG family members and TME in gliomas has not yet been elucidated. Based on The Cancer Genome Atlas (TCGA) database, we thus estimated the immune infiltration status of 22 immune cells in LGG and GBM, analyzed the relationship between NRGs and immune infiltration, and finally constructed a prognostic model of glioma, with the purpose of determining the distinct potential prognostic values of NRGs in gliomas. The results showed that NRGs are significantly related to the immune microenvironment in both LGG and GBM, and they also participate in m^6^A RNA methylation modification. Furthermore, NRG3 and NRG1 may serve as potential independent biomarkers in LGG and GBM in clinical applications, respectively, including glioma diagnosis and drug development.

## Materials and Methods

### Data Collection

Raw counts of RNA-seq data (level 3) with 510 LGG and 153 GBM samples from The Cancer Genome Atlas (TCGA) database (http://cancergenome.nih.gov/abouttcga), and 5 normal brain tissue samples from TCGA database and 2642 normal brain tissue samples from Genotype-Tissue Expression (GTEx, https://gtexportal.org/home/datasets) database were retrieved and used for the analysis of prognostic gene expression signatures and the construction of prognostic models in this study. Data preprocessing was carried out using R/Bioconductor package (v4.0.3, http://www.bioconductor.org), and all data retrieved from TCGA and GTEx were corrected and normalized using the “normalize between array” function of the “limma” R package. Clinical characteristics of LGG and GBM patients were shown in **Table 1**.

**Table 1:** Clinical characteristics of patients in TCGA-LGG and GBM dataset.

	Characters	LGG	GBM	Total
Status	Alive	385	31	416
	Dead	125	122	247
Age	Mean (SD)	42.9(13.4)	59.7(13.6)	46.8(15.2)
	Median [Min, Max]	41 [14, 87]	60 [21, 89]	46 [14, 89]
Gender	Female	228	54	282
	Male	282	99	381
Race	American Indian	1		1
	Asian	8	5	13
	Black	21	10	31
	White	470	137	607
Radiation therapy	Non-radiation	120		120
	Radiation	142	1	143

### Expression Level Analysis of NRG Family Members

We first analyzed the expression levels of NRG1-4 in brain tissues and nerve samples from GTEx database. And considering the tiny number of adjacent normal samples in TCGA database, we integrated the data of normal tissues in GTEx database to analyze the expression of NRG1-4 in gliomas and normal tissues through R software v4.0.3. For RNA-seq data, expression levels were TPM-normalized. The significance of the two groups of samples passed the Wilcox test. A p-value of less than 0.05 was considered statistically significant.

### Immune Estimations in LGG and GBM

For observing the differences of immune cells in LGG and GBM samples, we utilized an R package immunedeconv (30), through CIBERSORT algorithms, to make reliable immune infiltration estimations through integrating the TCGA and GTEx data. Meanwhile, considering that immune checkpoints are inhibitory regulatory molecules in the immune system, which are pivotally important to maintain self-tolerance, prevent autoimmune response, and minimize tissue damage by controlling the time and intensity of immune response, SIGLEC15, IDO1, CD274, HAVCR2, PDCD1, CTLA4, LAG3, and PDCD1LG2 were selected as immune-checkpoint–relevant transcripts and the expression values of these eight genes in LGG and GBM were extracted. In addition, GSCA (Gene Set Cancer Analysis) database (31) (http://bioinfo.life.hust.edu.cn/GSCA/#/) was used to estimate the correlation between NRG1-4 expression and immune infiltrates in LGG and GBM.

### Correlation Between the expression level of NRGs and Immune Infiltration Level in LGG and GBM

To comprehensively investigate whether the expression of NRGs was associated with immune infiltration level in LGG and GBM, we downloaded the data of six immune infiltrating cell types including B cell, CD4 T cell, CD8 T cell, neutrophils, macrophage and dendritic cell from the TIMER database (http://timer.cistrome.org/). Then, we further analyzed the correlation between NRGs expression and ImmuneScore, ESTIMATEScore and StromalScore of LGG and GBM calculated with R package ESTIMATED (32). Meanwhile, we analyzed the correlation between NRGs expression and tumor mutational burden (TMB)/microsatellite instability (MSI). In addition, we evaluated the relationship between NRGs expression and neoantigens counts in LGG and GBM, and investigated the expression relationship between NRGs and immune checkpoint genes. Spearman’s correlation analysis was used to depict the correlation, and a p-value of less than 0.05 was considered statistically significant.

### Correlation Between NRGs Expression and the m^6^A-Related Genes in LGG and GBM

To comprehensively investigate the expression distribution of the m^6^A-related genes and their correlation with NRGs expression in LGG and GBM, the m^6^A-related genes derived from research (33) on m^6^A modulators across 33 cancer types were retrieved and used for the analysis. The m^6^A-related genes include three types of regulators-related genes with methyltransferases (writers: METTL14, METTL3, RBM15, RBM15B, VIRMA, WTAP and ZC3H13), RNA binding proteins (readers: HNRNPA2B1, HNRNPC, IGF2BP1, IGF2BP2, IGF2BP3, RBMX, YTHDC1, YTHDC2, YTHDF1, YTHDF2 and YTHDF3), and demethylases (erasers: ALKBH5, FTO). Their correlation was evaluated using Spearman’s correlation analysis, and a p-value of less than 0.05 was considered statistically significant.

### Survival Analysis and Validation of the Prognostic Ability of NRGs

Survival analysis including overall survival (OS), progression-free survival (PFS), disease-specific survival (DSS) and disease-free survival (DFS) was used to evaluate the prognostic difference between LGG and GBM. The KM survival analysis for OS and PFS of NRGs in LGG and GBM with log-rank test was used to compare the survival difference. In the meantime, GSVA (Gene Set Variation Analysis) score on OS and PFS was performed through GSCA database to obtain comprehensive *NRG1-4* gene set variable analysis in LGG and GBM. To further investigate the prognostic value of NRGs in LGG and GBM, the forest plot was used to show the P value and hazard ratio (HR) with 95% confidence interval (CI) of NRGs through R package “forestplot” (http://www.bioconductot.org). Both univariate and multivariate Cox regression analysis were performed to construct the OS nomogram model through ‘rms’ R package. Based on the results of Cox proportional hazards analysis, the 1, 2, 3-year overall recurrence of patients with LGG and GBM can be predicted through nomogram model, which can be used to evaluate the risk of recurrence for patients by the points associated with each risk factor. In addition, the KM survival analysis with log-rank test were also used to compare the survival difference between high expression and low expression of NRGs in LGG and GBM, and time ROC analysis was performed to compare the predictive accuracy of gene and risk score.

### Functional Annotation and Enrichment Analysis

To further confirm the underlying functional annotation for gene sets associated with high expression and low expression of NRGs in LGG and GBM, Gene Ontology (GO) and Kyoto Encyclopedia of Genes and Genomes (KEGG) enrichment analysis in the present study were performed with P < 0.05 as the cutoff criteria through R package ClusterProfiler (34).

### GDSC Drug Sensitivity Analysis

To further investigate the drug sensitivity of NRG1-4 in pan-cancer, GSCA database was used to examine the effects of *NRG1-4* on drug response from the Genomics of Drug Sensitivity in Cancer (GDSC) database with the most extensive pharmacogenomic drug screening available.

### Statistical Analysis

Statistical analyses were performed using R software v4.0.3 (R Foundation for Statistical Computing, Vienna, Austria). P < 0.05 was considered statistically significant.

## Results

### Expression Analysis of NRGs in Normal Brain and Nerve Tissues and Different Grades of Gliomas in Human

**Figure 1:**
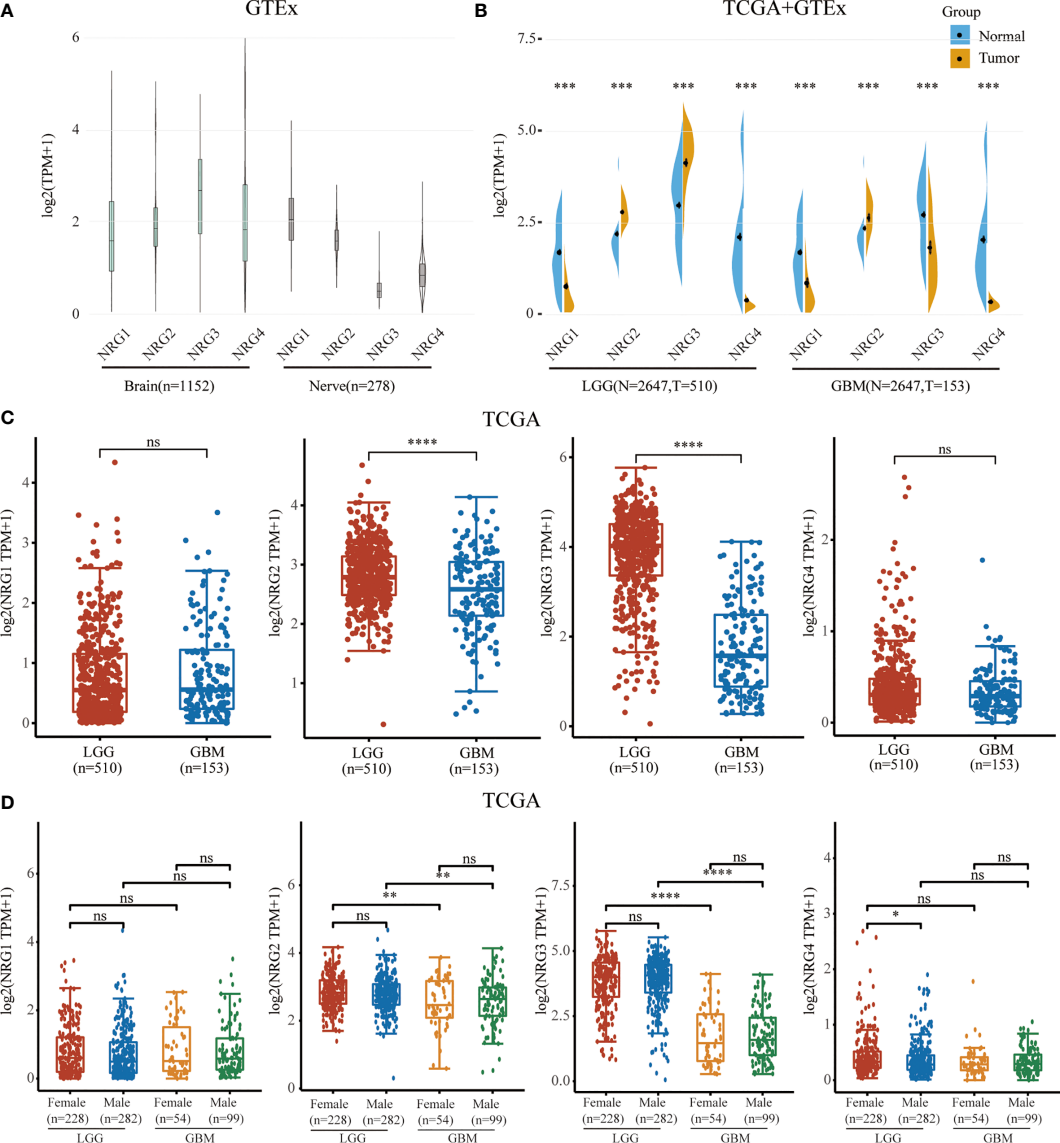
mRNA expression level of NRG family members. NRGs expression levels in the brain (n=1152) and nerve (n=278) from GTEx database **(A)**. NRGs expression levels in LGG (n=510), GBM (n=153) and normal tissues from GTEx (n=2642) and TCGA (n=5) database **(B)**. NRGs expression levels in LGG (n=510) and GBM (n=153) from TCGA database **(C)**. NRGs expression levels in patients of different genders with LGG (Female: n=228; Male: n=282) and GBM (Female: n=54; Male: n=99) **(D)**. The significance of the two groups of samples passed the Wilcox test. ns, no significance; *P < 0.05; **P < 0.01; ***P < 0.001; ****P < 0.0001.

We first identified the expression levels of NRG1, 2, 3 and 4 through TCGA and GTEx database. The GTEx database was used to compare NRG1, NRG2, NRG3 and NRG4 expression levels in normal human brain (n=1152) and nerve (n=278) tissues, which showed that all four NRG subtypes are abundantly expressed in the normal tissues (**Figure 1A**). Using the Wilcox test, we analyzed NRGs expression levels between normal (n=2647) and either LGG (n=510) or GBM (n=153) samples and found that the expression levels of four NRG subtypes in both LGG and GBM patients were significantly different from that in normal human tissues in TCGA and GTEx database (**Figure 1B**). Furthermore, we also found that only NRG2 and NRG3 were significantly differentially expressed in LGG compared to GBM, whereas there was no significance of NRG1 and NRG4 expression in LGG compared to GBM (**Figure 1C**). And only NRG4 expression level was significantly altered in LGG patients of different genders, compared to other NRG subtypes (**Figure 1D**). Based on the above data, it can be deduced that the gene expression of all four NRG subtypes is significantly changed in gliomas in comparison to normal samples.

### Landscape of the TME in LGG and GBM

**Figure 2:**
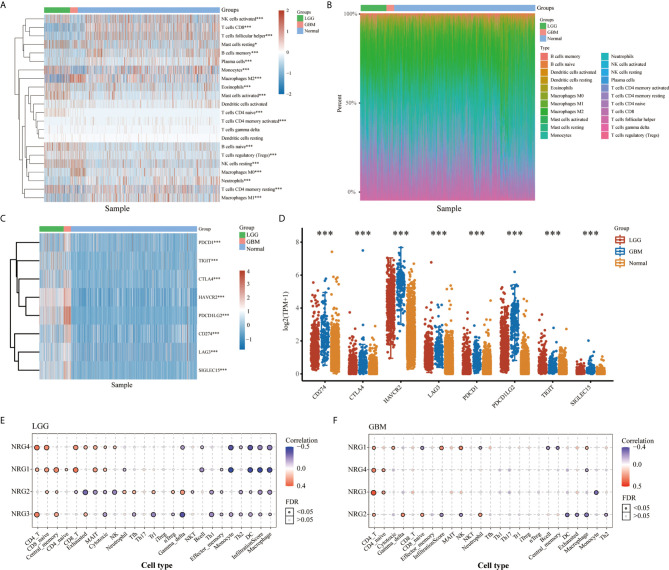
Landscape of the TME in LGG and GBM. The score heatmap of 22 immune cells in LGG and GBM, where different colors represent the expression trend in different samples **(A)** (LGG: n=510, GBM: n=153, Normal: n=5). The percentage abundance of tumor-infiltrating immune cells in each sample, with different colors representing different types of immune cells **(B)**. The abscissa represents the samples, and the ordinate represents the percentage of immune cell content in a single sample. The expression heatmap **(C)** and distribution **(D)** of immune checkpoints-related genes in LGG and GBM, where different colors represent the expression trend in different samples (LGG: n=510, GBM: n=153, Normal: n=2647). Correlation between NRG1-4 expression and immune infiltrates in both LGG **(E)** and GBM **(F)**. The significance of the different groups of samples passed the Kruskal-Wallis test. *P < 0.05; ***P < 0.001.

Cluster analysis revealed distinct patterns of immune cell infiltration in LGG and GBM. GBM exhibits high infiltration of CD4+ memory T cells, neutrophil cells, memory B cells, M0 macrophages, activated myeloid dendritic cells, regulatory T cells, activated NK cells, macrophage M2 and CD8+ T cells, whereas LGG shows significant increases in the infiltration of macrophage M1, follicular helper T cells, activated NK cells, monocyte, naive B cells, plasma B cells and naive CD4+ T cells (**Figures 2A, B**). In addition, expression distribution and heatmap analysis indicated that 8 immune checkpoint related genes, including SIGLEC15, IDO1, CD274, HAVCR2, PDCD1, CTLA4, LAG3, and PDCD1LG2, were differentially expressed in LGG and GBM compared to normal tissues. Among these genes, CTLA4, PDCD1LG2, CD274 and SIGLEC15 were significantly increased in GBM, and HAVCR2 was the most highly expressed in both LGG and GBM (**Figures 2C, D**). In the meantime, immune infiltrates analysis revealed consistently positive correlation between NRG1-4 expression and CD4 T cell, CD8 naive cell and central memory cell, and consistently negative correlation between NRG1-4 expression and Macrophage, infiltrationscore, DC, TH1, Th2, Monocyte, and effector memory cell in LGG (**Figure 2E**). However, it’s only observed that consistently positive correlation between NRG1-4 expression and CD4 T cell and CD4 naive cell exists in GBM (**Figure 2F**).

### The Correlation Between NRG Members and Immune Cell Infiltration

**Figure 3:**
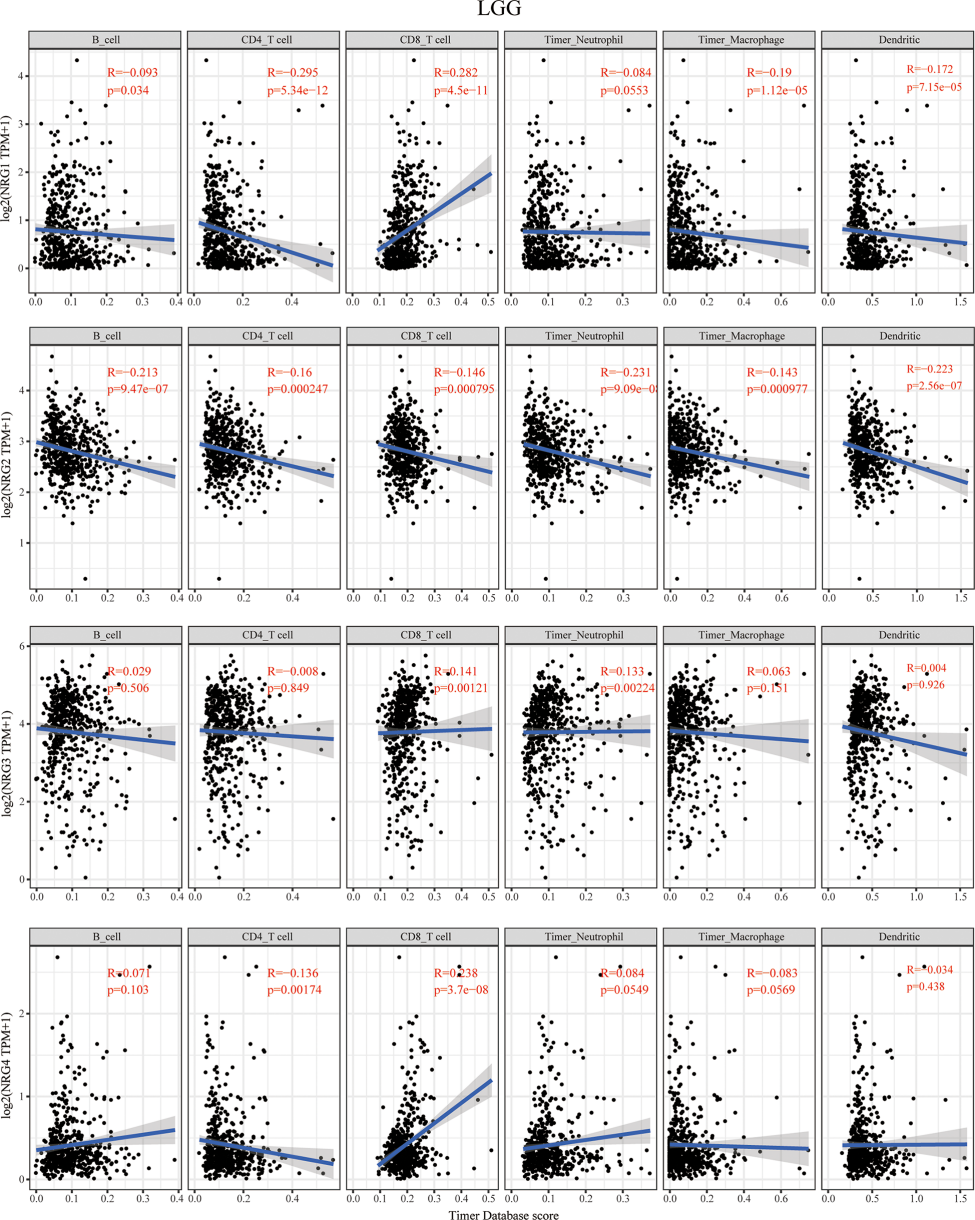
Correlations between NRGs expression and immune cell infiltration in LGG. Correlations between the abundance of 6 immune cells (B cell, CD4 T cell, CD8 T cell, neutrophils, macrophage and dendritic cell) from TIMER database and the expression of NRG1-4 in LGG. Spearman’s correlation analysis was used to describe the correlation. P < 0.05 was considered statistically significant.

**Figure 4:**
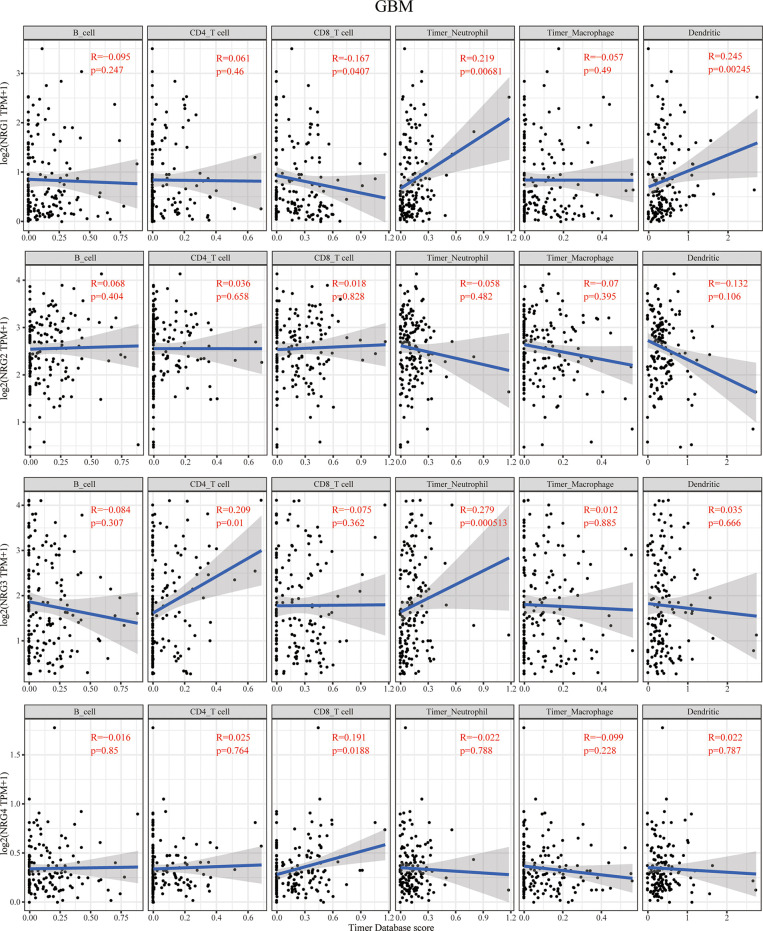
Correlations between NRGs expression and immune cell infiltration in GBM. Correlations between the abundance of 6 immune cells (B cell, CD4+ T cell, CD8+ T cell, neutrophils, macrophage and dendritic cell) from the TIMER database and the expression of NRG1-4 in GBM. Spearman’s correlation analysis was used to describe the correlation. P < 0.05 was considered statistically significant.

The TIMER database was then used to explore the correlation between NRG members and immune cell infiltration in LGG and GBM (**Figure 3** and **Figure 4**). The data of six kinds of immune infiltrating cells of LGG and GBM were downloaded from the TIMER database to analyze the correlation between the expression of four NRG genes and the scores of six types of immune infiltrating cells including B cells, CD4+ T cells, CD8+ T cells, neutrophil cells, macrophage cells and dendritic cells. Although expression of NRG1 was significantly negatively correlated with the infiltration of CD4+ T cells, macrophage cells and dendritic cells in LGG, it is distinctly in a significant positive correlation with that of CD8+ T cells. NRG2 was negatively associated with the infiltration of all six immune cells in LGG. The expression of NRG3 was positively associated with the infiltration of CD8+ T cells and neutrophil cells in LGG. NRG4 expression showed a significant correlation with the infiltration of the CD4+ T cells and CD8+ T cells in LGG (**Figure 3**).

The context of immune cell infiltration of in correlation to four NRG members in GBM is quite different from those in LGG. NRG1 expression was negatively correlated with the infiltration of CD8+ T cells and obviously positively correlated with that of neutrophils and dendritic cells in GBM. In contrast to that in LGG, no immune cell infiltration cells were found to be significantly correlated with NRG2 expression in GBM. The infiltration of CD4+ T cells and neutrophils is distinctly in a significant positive correlation with NRG3 expression in GBM. NRG4 expression was shown to have only correlated with the infiltration of CD8+ T cells in GBM (**Figure 4**).

### NRGs Members Expression Are Significantly Associated With Immune Scores, Stromal Scores, and ESTIMATE Scores in LGG and GBM

**Figure 5:**
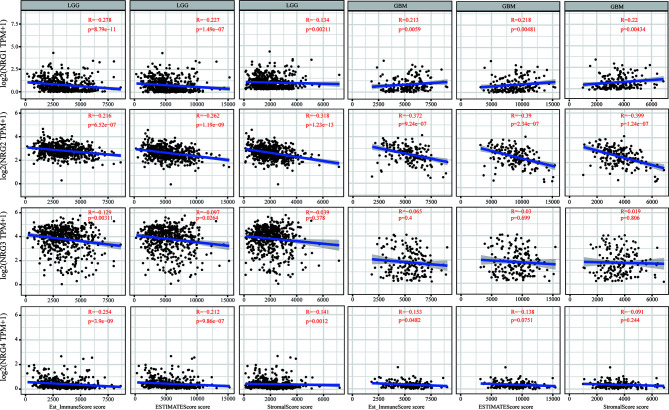
Immune scores of NRGs in patients with LGG and GBM. Correlations between the immune score (ImmuneScore, ESTIMATEScore, and StromalScore) and the expression of NRG1-4 in LGG and GBM. Spearman’s correlation analysis was used to describe the correlation. P < 0.05 was considered statistically significant.

We further conducted the ESTIMATE algorithm to obtain the immune scores, stromal scores and ESTIMATE scores based on expression data from TCGA database to investigate whether NRGs members expression is related to the level of immune invasion and the level of infiltrating stromal and immune cells in LGG and GBM samples. As shown in **Figure 5**, the analysis result revealed that the expression levels of NRG1, NRG2 and NRG4 are all significantly negatively correlated with immune scores, stromal scores and ESTIMATE scores (P < 0.01 for all scores) in LGG. The expression level of NRG3 is negatively correlated with immune scores (P < 0.01) and ESTIMATE scores (P < 0.05). On the contrary, NRG1 expression is positively correlated with immune scores, stromal scores and ESTIMATE scores (P < 0.01 for all scores) in GBM, suggesting that NRG1 plays opposite roles in different grades of gliomas. In line with that in LGG the expression of NRG2 expression is negatively correlated with all the three scores (P < 0.01 for all scores) in GBM. The expression of NRG4 was negatively correlated with the immune score (P < 0.05). However, there was no significant correlation between NRG3 expression and three types of scores in GBM.

### The Correlation Between the Expression Level of NRGs and TMB/MSI

**Figure 6:**
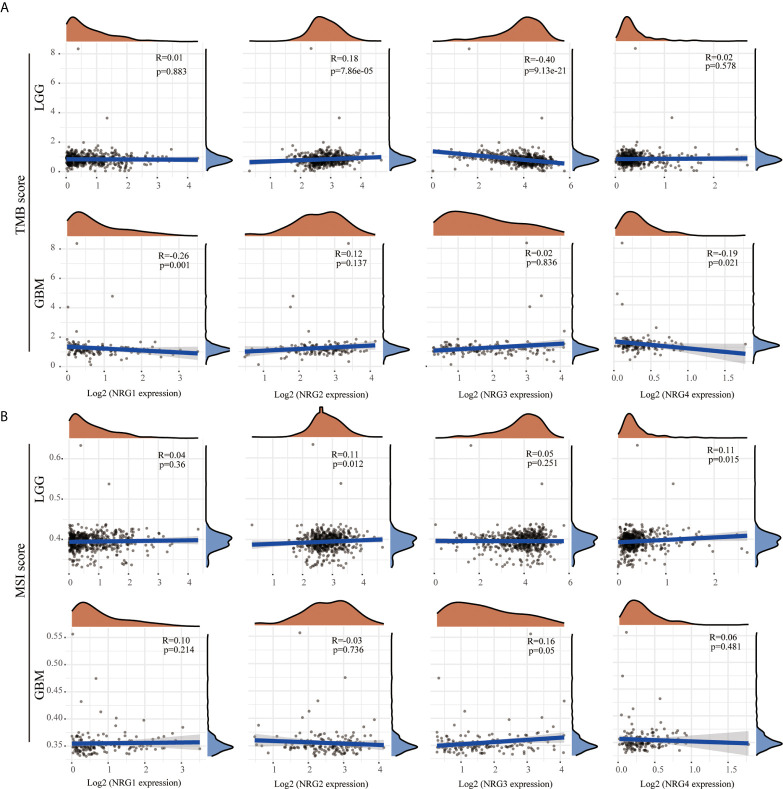
Correlations between NRGs expression and TMB/MSI in LGG and GBM. Correlation analysis of NRG1-4 expression and TMB **(A)** /MSI **(B)** in LGG and GBM. The horizontal axis represents the expression distribution of NRG1-4, and the ordinate represents the expression distribution of TMB/MSI score. The density curve on the right represents the distribution trend of TMB/MSI score; the upper density curve represents the distribution trend of NRG1-4. Spearman’s correlation analysis was used to describe the correlation. P < 0.05 was considered statistically significant.

Although TMB and MSI are prognostic biomarkers for many cancer types, their prognostic value in LGG and GBM remains unclear. By using multi-omics data from TCGA, we systematically analyzed the correlations between TMB/MSI and NRG subtypes expression level in gliomas to identify the influence of NRGs in the development and therapy of LGG and GBM. The results showed that NRG2 expression was positively correlated with TMB (P < 0.01), whereas that of NRG3 was negatively correlated with TMB in LGG (P < 0.01) (**Figure 6A**). The expression of both NRG1 and NRG4 were negatively correlated with TMB in GBM (P < 0.01 for NRG1, and P < 0.05 for NRG4) (**Figure 6A**). The expression of both NRG2 and NRG4 was positively correlated with MSI in LGG (P < 0.05 for both) (**Figure 6B**), whereas only NRG3 expression was positively correlated with MSI in GBM (P = 0.05) (**Figure 6B**).

### The Correlation Between NRGs Expression and Neoantigens in LGG and GBM

**Figure 7:**
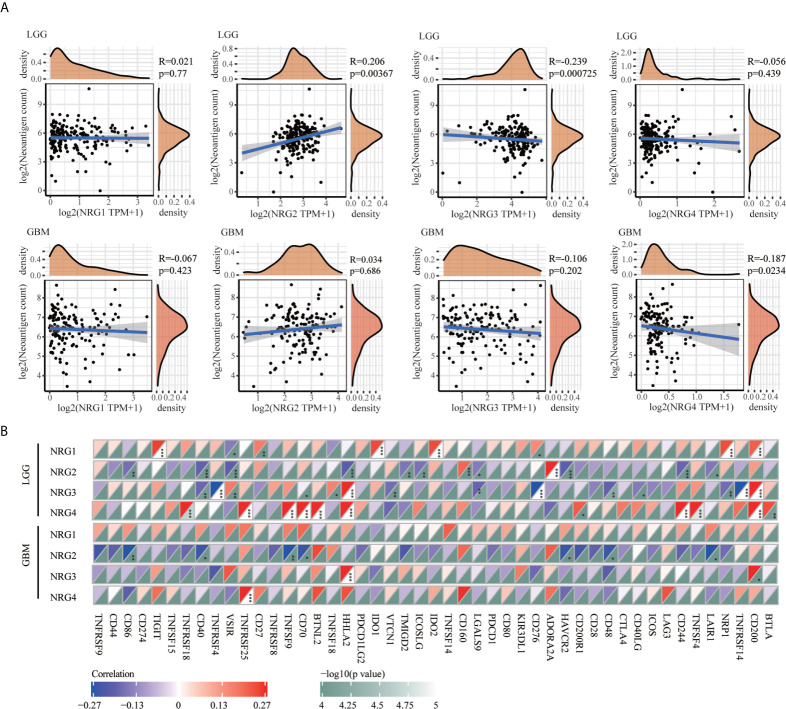
Correlations between NRGs expression and neoantigens/immune checkpoint in LGG and GBM. Correlation analysis of NRG1-4 expression and neoantigen counts in LGG and GBM **(A)**. The horizontal axis represents the expression distribution of NRG1-4, and the ordinate is the distribution of neoantigen counts. The density curve on the right represents the distribution trend of neoantigen counts; the upper density curve represents the distribution trend of NRG1-4. Correlations between immune checkpoint-related genes and the expression of NRG1-4 in LGG and GBM **(B)**. Spearman’s correlation analysis was used to describe the correlation. *P < 0.05; **P <0.01; ***P < 0.001.

Because of evolved mechanisms to escape immune control in cancer, neoantigens (wrong protein produced after tumor somatic mutation) and immune checkpoint genes are considered important targets of checkpoint blockade therapy for immunotherapy of cancer. As shown in **Figure 7A**, a significantly positive correlation was found between NRG2 and neoantigens count (P < 0.01), and a significantly negative correlation was found between NRG3 and neoantigens count (P < 0.01) in LGG. Only the expression of NRG4 was found to be significantly negatively correlated with neoantigens in GBM (P < 0.05) (**Figure 7A**). Correspondingly, the expression of NRG2, NRG3 and NRG4 was significantly associated with more immune checkpoint genes in LGG than in GBM (**Figure 7B**). Different from that in GBM, NRG2 was negatively associated with the expression of VSIR, HHLA2, TMIGD2, ICOSLG, LGALS9, HAVCR2 and CD244, but was positively associated with CD160 and ADORA2A in LGG. However, no significant correlations between NRG2 and these above genes were found in GBM. NRG3 expression was negatively correlated with CD40, TNFRSF4, CD70, VTCN1, LGASL9, CD276, CD48, CD40LG, NRP1 and TNFRSF14, and was positively correlated with VSIR and TNFSF18 in LGG. However, no significant correlations between NRG3 and these above genes were found in GBM. In addition, NRG4 expression was in direct proportion to genes TNFSF18, TNFRSF25, RNFSF9, CD70, BTNL2, CD200R1, CD244, TNFSF4 and BTLA. However, no direct proportion was found between NRG3 and these above genes in GBM.

### The Association of NRG Family Members With the Expression Distribution of m^6^A-Related Genes in LGG and GBM

**Figure 8:**
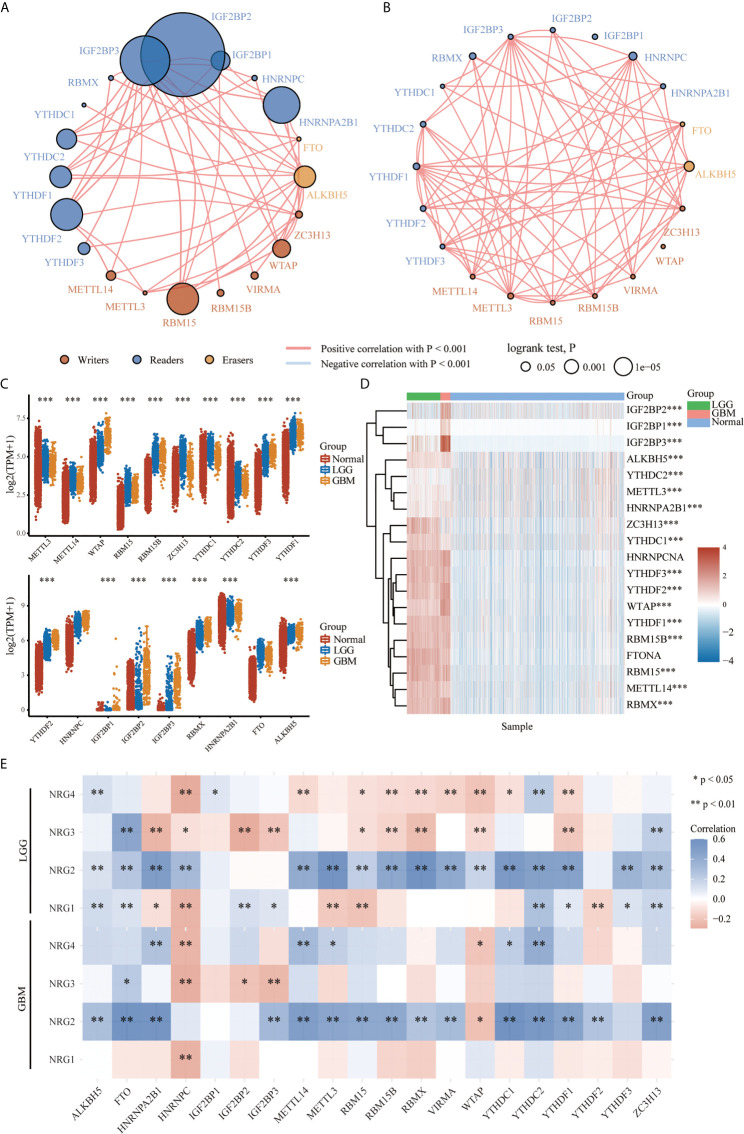
The expression distribution of the m^6^A-related genes in LGG and GBM. Correlation network analysis of the m^6^A-related genes in LGG **(A)** and GBM **(B)**. The circle represents the m^6^A-related genes, and the line represents the relationship between genes. The red represents the positive correlation and the blue represents the negative correlation, with lines of different thickness representing the extent of correlation between two genes. Larger circles represent higher prognosis log-rank p, with the brown, blue and orange circles representing writers, readers, and erasers, respectively. Expression level of m^6^A-related genes in LGG and GBM **(C)**, and expression distribution heatmap of m^6^A-related genes in LGG and GBM, where different colors represent the expression trend in different samples (LGG: n=510, GBM: n=153, Normal tissue: n=2647) **(D)**. Correlations between m^6^A-related genes and the expression of NRG1-4 in LGG and GBM **(E)**. The significance of the different groups of samples passed the Kruskal-Wallis test, and Spearman’s correlation analysis was used to describe the correlation. *P < 0.05; **P < 0.01; ***P < 0.001.

To further identify the molecular mechanisms by which the four NRG members are involved in m^6^A regulators of different grades of gliomas, we first examined the protein-protein interaction (PPI) correlation network among m^6^A regulators in LGG (**Figure 8A**) and GBM (**Figure 8B**), the expression of individual m^6^A regulators in LGG and GBM (**Figure 8C** and **Figure 8D**), and the correlations between m^6^A-related genes and the expression of NRG1-4 in LGG and GBM (**Figure 8E**). RNA methylation is regulated by different types of regulators, including methyltransferases (“writers”), RNA binding proteins (“readers”) and demethylases (“erasers”), whose roles exist in collaboration in the context of cancer. The PPI among the m^6^A RNA methylation regulators were further analyzed to better understand the function of these regulators in the pathogenesis of LGG and GBM. As shown in **Figure 8A** and **Figure 8B**, these writers, erasers, and readers were positively correlated with each other frequently to varying extents (P<0.001). The results suggested higher correlations among IGF2BP2, IGF2BP1, IGF2BP3, HNRNPA2B1, YTHDC1, YTHDC2, YTHDF1, YTHDF2, YTHDF3, RBM15, WTAP and ALKBH5 in LGG. Except for IGF2BP1 and WTAP, significant correlations among other 18 genes were observed in GBM. The expression level of 17 m^6^A-related genes in normal, LGG and GBM patients was significantly different, and the expression level of METTL14, WTAP, RBM15, RBM15B, ZC3H13, YTHDC1, YTHDF3, YTHDF1, YTHDF2, IGF2BP2, IGF2BP3, RBMX, HNRNPA2B1 and ALKBH5 in the patients with LGG (P<0.001) and GBM (with P<0.001) were significantly higher than that in the patients of normal group (**Figure 8C**). It is worth noting that the expression of IGF2BP1, IGF2BP2 and IGF2BP3 in GBM was significantly higher than that in the normal patients in the heatmap (P < 0.01 for all three genes) (**Figure 8D**). Next, we found that the expression of a majority of m^6^A genes expression was obviously correlated with that of NRG1-4 in LGG. However, only HNRNPC expression was found to be negatively correlated with that of NRG1, suggesting that NRG1-4 were partly involved in the RNA methylation molecular mechanism in LGG and GBM.

### Prognostic Value of the NRGs in LGG and GBM

**Figure 9:**
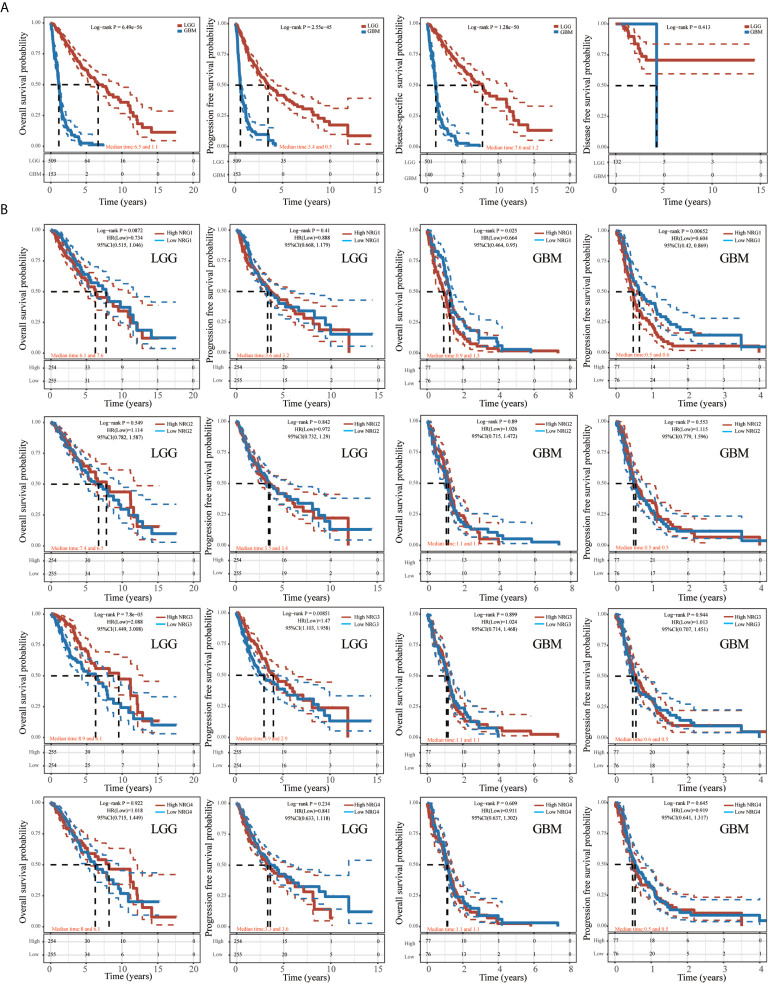
Kaplan-Meier survival analysis of NRGs in LGG and GBM. Kaplan-Meier survival analysis of LGG and GBM, including overall survival (OS), progression-free survival (PFS), disease-specific survival (DSS) and disease-free survival (DFS) **(A)**. Kaplan-Meier survival analysis of NRG1-4 in LGG and GBM, including OS and PFS **(B)**.

**Figure 10:**
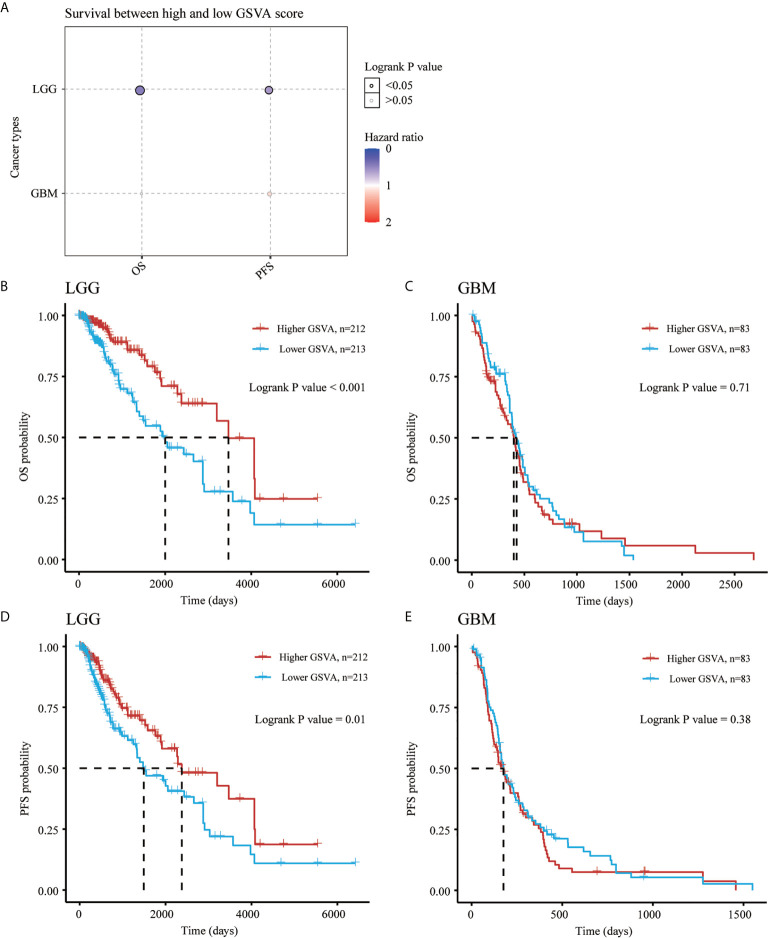
Gene Set Variation Analysis of NRGs in LGG and GBM. Survival between high and low GSVA score in both LGG and GBM **(A)**. OS probability analysis for high and low GSVA scores of NRG1-4 in LGG **(B)** and GBM **(C)**. PFS probability analysis for high and low GSVA scores of NRG1-4 in LGG **(D)** and GBM **(E)**.

As shown in **Figure 9**, the K-M curve showed significantly lower overall survival probability (OS), Progression-Free Survival (PFS) and Disease-specific survival (DSS) in GBM than in LGG (P < 0.001) (**Figure 9A**). We then performed Kaplan-Meier analysis to investigate the impact of genetic alterations of NRG1-4 on OS and PFS in patients with LGG and GBM. Patients with high NRG1 expression exhibit significantly shorter PFS than those with lower NRG1 expression in GBM patients (HR, 0.604; 95% CI, 0.42–0.869; P < 0.01) (**Figure 9B**). In addition, patients with high NRG3 expression show significantly higher OS and PFS than those with low NRG3 expression in LGG (P<0.001 for OS, and P<0.01 for PFS) (**Figure 9B**). In addition, GSVA score revealed that NRG1-4 gene set are more related to survival in LGG compared with that in GBM (**Figure 10A**). And higher GSVA showed a better prognosis of OS (P<0.001) and PFS (P=0.01) in LGG (**Figures 10B, C**) than OS (P<0.71) and PFS (P=0.38) in GBM (**Figures 10D, E**). Meanwhile, through CGGA database (Chinese Glioma Genome Atlas; http://www.cgga.org.cn/), we also evaluated the prognostic value of NRG family members in both primary and recurrent gliomas in different WHO grades. The results showed that NRG1 (p=0.0045), NRG3 (p=0.0073) and NRG4 (p=0.026) were significantly related to survival in recurrent gliomas (all WHO grade, **Figures S1**, **S3**, **S4**), and NRG2 (p=0.031) and NRG3 (p<0.0001) were significantly related to survival in primary gliomas (all WHO grades, **Figures S2**, **3**).

**Figure 11:**
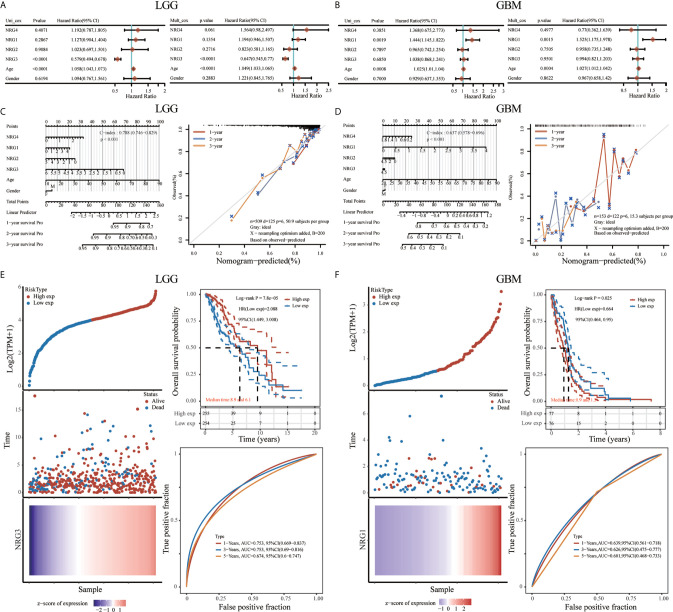
Prognostic value of NRGs in LGG and GBM. Univariate and multivariate Cox regression of NRG1-4 in LGG **(A)** and GBM **(B)**. Nomogram to predict the 1, 2 and 3-year overall survival of patients with LGG **(C)** and GBM **(D)**. The dashed diagonal line represents the ideal nomogram, and the blue, red and orange lines represent the 1, 2 and 3-year observed nomograms, respectively. Risk model and prognostic analysis of NRG3 in LGG **(E)**, and NRG1 in GBM **(F)**, respectively. The prognostic risk model shows the Risk type (top left), patient status (middle left) and mRNA expression heatmap (bottom left), and Kaplan–Meier curves of OS (top right) and time-dependent ROC (bottom right) for NRG3 in LGG **(E)** and NRG1 in GBM **(F)**.

To further confirm the prognostic value of different NRG family members signature in LGG and GBM, both univariate and multivariate Cox regression analyses were performed, showing that only NRG3 is independently associated with the OS of LGG patients (**Figure 11A**), and only NRG1 is significantly related to theOS of GBM patients (**Figure 11B**). With the inclusion of clinical relevance and prognostic value of age and gender, a prognostic nomogram based on different NRG subtypes expression signature, age and gender were established as a clinically dependable predictive method for predicting the survival probability of 1-, 2-, and 3-year survival of patients with LGG (**Figure 11C**) or GBM (**Figure 11D**). The C-index of the nomogram is 0.788 (95% CI, 0.746 to 0.829; P<0.001) in Fig.11C and 0.637 (95% CI, 0.578 to 0.696; P<0.001) in Fig.11D, respectively. Furthermore, to evaluate the predictive efficiency of NRG3 expression level in the 1, 3, and 5-year survival rate in LGG, we performed ROC curve utilizing the data from TCGA datasets. AUC was 0.753 at 1-year stage (95% CI, 0.669 to 0.837), 0.753 at 3-year stage (95% CI, 0.69 to 0.816), and 0.674 (95% CI, 0.6 to 0.747) at 5-year stage, respectively, suggesting the appreciable reliability of NRG3 as biomarker for LGG prognosis (**Figure 11E**). Similarly, **Figure 11F** also revealed a favorable predictive value for 1-, 3-, and 5-years OS rates, with AUC values of 0.639 (95% CI, 0.561 to 0.718), 0.626 (95% CI, 0.475 to 0.777) and 0.626 (95% CI, 0.468 to 0.733), respectively, supporting the efficacy of NRG1 as a biomarker for GBM prognosis (**Figure 11F**).

### Functional Enrichment for Gene Set Associated With High/Low Expression of NRG3 in Patients With LGG and With High/Low Expression of NRG1 in Patients With GBM

**Figure 12:**
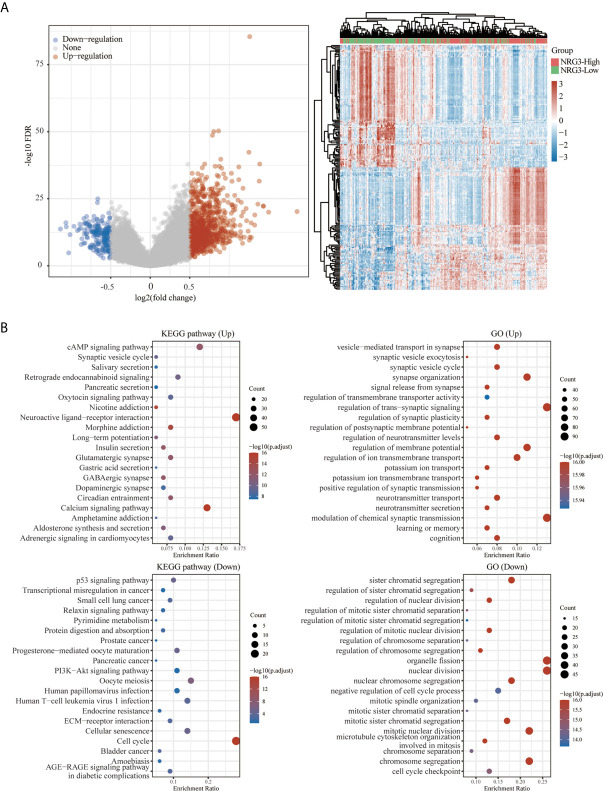
Functional enrichment for gene set associated with high/low expression of NRG3 in patients with LGG. Differentially expressed genes for high expression of NRG3 vs low expression of NRG3 in LGG were shown in the volcano plot **(A)**, with blue dots representing significantly down-regulated genes and orange dots representing significantly up-regulated genes in high expression of NRG3 in LGG, and heatmap exhibits the expression level. Enrichment analysis for KEGG pathway and GO term of down-regulated genes and up-regulated genes in high expression of NRG3 in LGG **(B)**.

**Figure 13:**
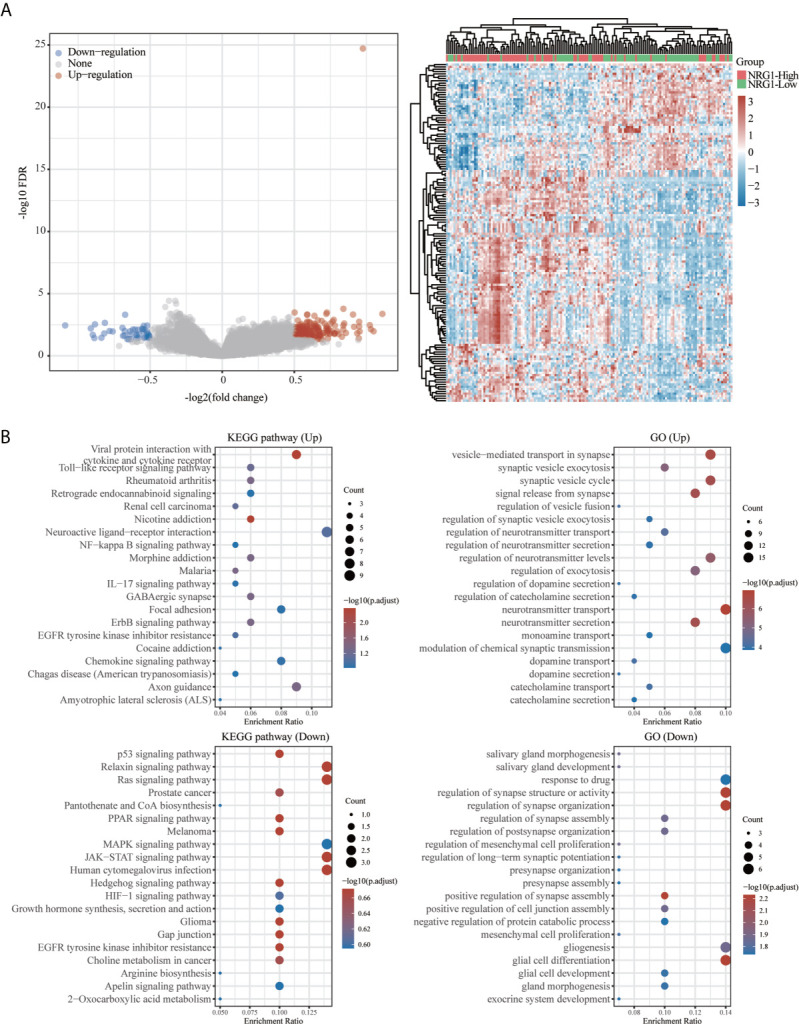
Functional enrichment for gene set associated with high/low expression of NRG1 in patients with GBM. Differentially expressed genes for high expression of NRG1 vs low expression of NRG1 in GBM were shown in the volcano plot **(A)**, with blue dots representing significantly down-regulated genes and orange dots representing significantly up-regulated genes in GBM with high expression of NRG1. The heatmap exhibits the expression level. Enrichment analysis for KEGG pathway and GO term of down-regulated genes and up-regulated genes in GBM with high expression of NRG1 **(B)**.

A total of 663 glioma samples (510 LGG samples and 153 GBM samples) and 2647 normal samples from TCGA and GTEx database were analyzed. In order to further determine the function of NRG3 in LGG and NRG1 in GBM, we analyzed the KEGG pathway and GO term enrichment of the high/low expression of NRG3 in patients with LGG (**Figure 12**) and high/low expression of NRG1 in patients with GBM (**Figure 13**), respectively. We first analyzed the differentially expressed genes (DEGs) with “adjusted P < 0.05 and | Log2 (Fold Change) | >0.5” by using Limma R package (version: 3.40.2) of R software. There are 1117 up-regulated DEGs and 318 down-regulated DEGs in the comparison of LGG samples with high expression of NRG3 and those with low expression of NRG3 (**Figure 12A**), and 431 up-regulated DEGs and 90 down-regulated DEGs in the comparison of GBM samples with high expression of NRG1 and those with low expression of NRG1 (**Figure 13A**).

To explore the function of the up- and down-regulated DEGs, an enrichment analysis was carried out on GO term and KEGG pathways using R package ClusterProfiler (34). As shown in **Figure 12B**, KEGG analysis revealed up-regulated-DEGs in two groups enriched mainly in the cAMP signaling pathway, neuroactive ligand-receptor interaction and calcium signaling pathway, and down-regulated DEGs in two groups enriched mainly in cell cycle. GO term analysis revealed that the up-regulated DEGs in two groups are enriched mainly in the synapse organization, regulation of trans-synaptic signaling, regulation of membrane potential, regulation of ion transmembrane transport and modulation of chemical synaptic transmission, while the down-regulated-DEGs in two groups are enriched mainly in sister chromatid segregation, organelle fission, nuclear division, mitotic nuclear division and chromosome segregation.

As shown in **Figure 13B**, KEGG pathway analysis showed that up-regulated DEGs in two groups were enriched mainly in viral protein interaction with cytokine and cytokine receptor and the down-regulated DEGs in two groups are enriched mainly in relaxin signaling pathway, ras signaling pathway, JAK-STAT signaling pathway and human cytomegalovirus infection. GO analysis revealed that up-regulated DEGs in two groups are enriched mainly in the vesicle-mediated transport in synapse, synaptic vesicle cycle, neurotransmitter transport and neurotransmitter secretion and the down-regulated DEGs in two groups are enriched mainly in regulation of synapse structure or activity, regulation of synapse organization and glial cell differentiation.

### Correlation Between NRG1-4 Expression and GDSC Drug Sensitivity in Pan-Cancer

**Figure 14:**
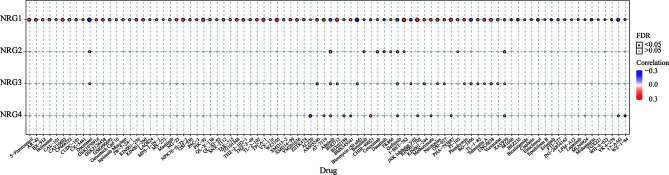
GDSC drug sensitivity analysis. Correlation between NRG1-4 expression and GDSC drug sensitivity in pan-cancer.

The correlation between NRG1-4 expression and drug sensitivity in pan-cancer was shown in **Figure 14**, and the drug responses may aid in drug repositioning and new drug development. The results showed that NRG1 was most associated with GDSC drug response, and only a few drugs were related to NRG2-4, such as Docetaxel, Afatinib, Gefitinib and XAV939. The results may contribute to develop new therapeutic targets for the clinical treatment of gliomas.

## Discussion

Gliomas are the most popular tumors of the central nervous system (35), in which LGG is one of the prevalent and infiltrative types of primary malignant intracranial tumor type (36), and GBM is considered as the most common and aggressive form (37). Accurate prognostic evaluation is essential for the appropriate treatment decisions at early stage to improve patient outcomes. Cancer treatment based on biomarkers can improve prognosis estimates for some malignant tumor. Thus, the pathogenesis-based discovery of crucial glioma biomarkers may contribute to the diagnosis and treatment of gliomas. Tumor microenvironment (TME) has in recent years gained extensive attention for their important roles in the occurrence and development of tumors (38, 39). Given the role of NRGs in the development of nervous system (11, 40, 41), it will thus be very interesting to further explore the more exact function of NRG family members in gliomas. In this study, we comprehensively analyzed the role of NRGs in gliomas from expression level, tumor immune microenvironment, m^6^A modification and prognostic value based on the TCGA database.

Our findings showed that NRGs are abundantly expressed in both brain and nerve tissues, and are differentially expressed in gliomas and normal tissues (**Figure 1**). We also found the significant differences of various immune cells and differential expression of immune checkpoint between gliomas and normal tissues (**Figure 2**). Immune cell infiltration analysis demonstrated that NRG family members are significantly associated with different immune cells, including B cell, CD4 T cell, CD8 T cell, neutrophils, macrophage and dendritic cell, in both LGG and GBM, especially with CD8 T cell among 6 immune cells in LGG (**Figures 3**, **4**). Intriguingly, there are some differences in the results of immune infiltration correlation between GSCA database and TIMER database. For example, NRG1 was positively correlated with the infiltration of CD4+ T cell in GSCA database, but significantly negatively correlated with the infiltration of CD4+ T cell in TIMER database in LGG, which may be due to the inconsistent data processing of different database. This is a very interesting phenomenon, but it can be determined that NRG family members are involved in the infiltration of most immune cells, which need to be verified by further experiments in the future. In addition, we also found that NRGs were significantly correlated with TME by the immune score. As an important part of TME, immune cell infiltration at the primary lesion is involved in tumor initiation and malignant progression including tumor-associated angiogenesis, tumor cell invasion and migration, as well as antitumor immune response (42, 43). And previous studies showed that the ErbB receptors of NRG family member were significantly involved in TME in cancers (44, 45). Thus, it is also worth exploring how the interaction between NRGs and their ErbB receptors to determine tumor progression and patient survival in gliomas in following studies. Some evidence indicated that nerves play roles in the TME, which significantly influences tumor progression and may serve as an alternative route for the dissemination of tumor cells (46, 47). In the meantime, tumor cells can develop surrounding existing nerves and eventually invade them, known as perineural invasion (PNI) (48). Furthermore, PNI has been observed in various diseases, causing severe pain in patients and leading to poor prognosis (49). Considering that NRG family members are abundantly expressed in nerves and significantly involved in TME in the present study, NRGs may thus alter the TME status through immune infiltration in gliomas.

TMB and MSI have gained more and more attention in immunotherapy, which play an essential role in TME and serve as biomarkers for immunotherapy prediction (50, 51). The TMB score analysis revealed that NRG2 and NRG3, and NRG1 and NRG4 were significantly correlated with TMB in LGG and GBM, respectively. However, only NRG2 and NRG4 are conspicuously correlated with MSI in LGG (**Figure 6**). Under normal circumstances, the immune system can recognize and remove tumor cells in the TME. However, for survival and development, tumor cells can adopt different strategies to suppress the immune system, so as to survive in various stages of anti-tumor immune response (52). Thus, neoantigens play a vital role in immunotherapy (53). As is shown in **Figure 7**, the expression levels of NRG2 and NRG3 in LGG, and NRG4 in GBM are significantly associated with neoantigen counts, indicating that NRGs may contribute to the clinical immunotherapy. In addition, recent research showed that m^6^A RNA methylation can regulate the self-renewal and tumorigenesis of glioblastoma stem cells (54), and m^6^A demethylase ALKBH5 can maintain tumorigenicity of glioblastoma stem-like cells by sustaining FOXM1 expression and cell proliferation program (55). Our investigation of m^6^A-related genes in gliomas revealed that RNA binding proteins-related genes (Readers) are more significantly involved in the stability, translation efficiency, alternative splicing and localization of mRNA in LGG when compared with GBM, and NRG family members are prominently correlated with various m^6^A-related genes in gliomas (**Figure 8**), which indicate multiple potentially molecular functions of NRGs in m^6^A RNA modification in gliomas.

Previous study showed that some genes were significantly related to prognosis and may be potential biomarkers for gliomas, such as NUSAP1 (56), GINS2 (57), TRIM21 (58), IL-6 (59) and LATS1 (60). However, the potential therapeutic targets in the treatment of gliomas are still elucidated. Thus, accurate survival prediction through comprehensive indicators is also essential for glioma patients. Our Kaplan–Meier survival analysis revealed that LGG patients have a better prognosis than GBM patients, and NRG1 and NRG3 expression is significantly associated with prognosis, including OS and PFS. The high NRG3 expression is significantly associated with a good prognosis (p=7.8e-05, HR=2.088, 95%CI: 1.449, 3.008 for OS, and p=0.00851, HR=1.47, 95%CI: 1.103, 1.958 for PFS) for patients with LGG, but the high NRG1 expression is significantly associated with a poor prognosis (p=0.025, HR=0.664, 95%CI: 0.464, 0.95 for OS, and p=0.00652, HR=0.604, 95%CI: 0.42, 0.869 for PFS) for patients with GBM. These findings indicated that high NRG3 expression may inhibit the malignant progression of LGG as a protective factor for LGG patients, while high NRG1 expression may promote the development of GBM as one of the risk factors. In addition, GSVA score showed that NRG family members are more significantly associated with prognosis in LGG compared with GBM (**Figure 10**). Through application of ROC curve and construction of the Nomogram model, we also demonstrated the predictive value of NRG family members in OS in both LGG and GBM, further confirming the efficacy of NRG1 and NRG3 as indicators for prognosis of GBM and LGG, respectively. Meanwhile, the results of Cox regression analysis also indicated that NRG3 and NRG1 can serve as an independent prognostic biomarker for LGG and GBM, respectively (**Figure 11**). Furthermore, functional enrichment analysis showed that gene sets associated with NRG3 in LGG are mainly enriched in neuroactive ligand-receptor interaction, cell cycle, calcium signaling pathway, and cAMP signaling pathway (**Figure 12**), and gene sets associated with NRG1 in GBM were mainly involved in viral protein interaction with cytokine and cytokine receptor, relaxin signaling pathway, ras signaling pathway, JAK-STAT signaling pathway, and human cytomegalovirus infection and MAPK signaling pathway (**Figure 13**).

In conclusion, we comprehensively analyzed the differential expression and immune infiltration of NRG family members in LGG and GBM, and evaluated their clinical and prognostic values. Our results showed that the expression levels of NRG1 and NRG4 are significantly reduced in LGG and GBM tissues, whereas NRG2 is remarkably elevated. However, the expression level of NRG3 is increased in LGG, but decreased in GBM. Immune score revealed that NRG family members are significantly related to the immune microenvironment and correlated with immune cell infiltration, especially the CD8+ T cell in LGG and GBM, indicating that NRGs may contribute to altered immune status. The prognostic model analysis showed that NRG3 and NRG1 can serve as potential independent biomarkers in LGG and GBM, respectively. Further investigation into the molecular functions of NRG family members in gliomas may provide a better understanding on future treatment strategies. Furthermore, more clinical factors for achieving higher accuracy in predictions of glioma prognosis should also be considered in both clinical and experimental studies.

## Data Availability Statement

Publicly available datasets were analyzed in this study. This data can be found here: TCGA: http://cancergenome.nih.gov/abouttcga.

## Author Contributions

Conceived and designed the study: W-jZ and G-yO. Performed and analyzed the data: W-jZ and G-yO. Wrote the paper: G-yO, and W-wL drafted the paper. Edited the paper: W-jZ edited and finalized the submission version of the manuscript. All authors contributed to the article and approved the submitted version.

## Funding

This work was supported by the National Natural Science Foundation of China (81471279 and 81171138 to W-jZ). Research start-up fund of Jiangnan University (1285081903200020 to W-jZ). Research start-up fund of Wuxi School of Medicine, Jiangnan University (1286010242190060 to W-jZ).

## Conflict of Interest

The authors declare that the research was conducted in the absence of any commercial or financial relationships that could be construed as a potential conflict of interest.

## Supplementary Material

Supplementary Figure 1 The prognostic value of NRG1 in primary and recurrent gliomas in different WHO grades.

Supplementary Figure 2 The prognostic value of NRG2 in primary and recurrent gliomas in different WHO grades.

Supplementary Figure 3 The prognostic value of NRG3 in primary and recurrent gliomas in different WHO grades.

Supplementary Figure 4 The prognostic value of NRG4 in primary and recurrent gliomas in different WHO grades.
