# Supplementary figures and images for: Integrative Analysis of Neuregulin Family Members-Related Tumor Microenvironment for Predicting the Prognosis in Gliomas
Source: Front Immunol. 2021 May 13;12:682415. doi: 10.3389/fimmu.2021.682415 (PMC8155525; doi:10.3389/fimmu.2021.682415)

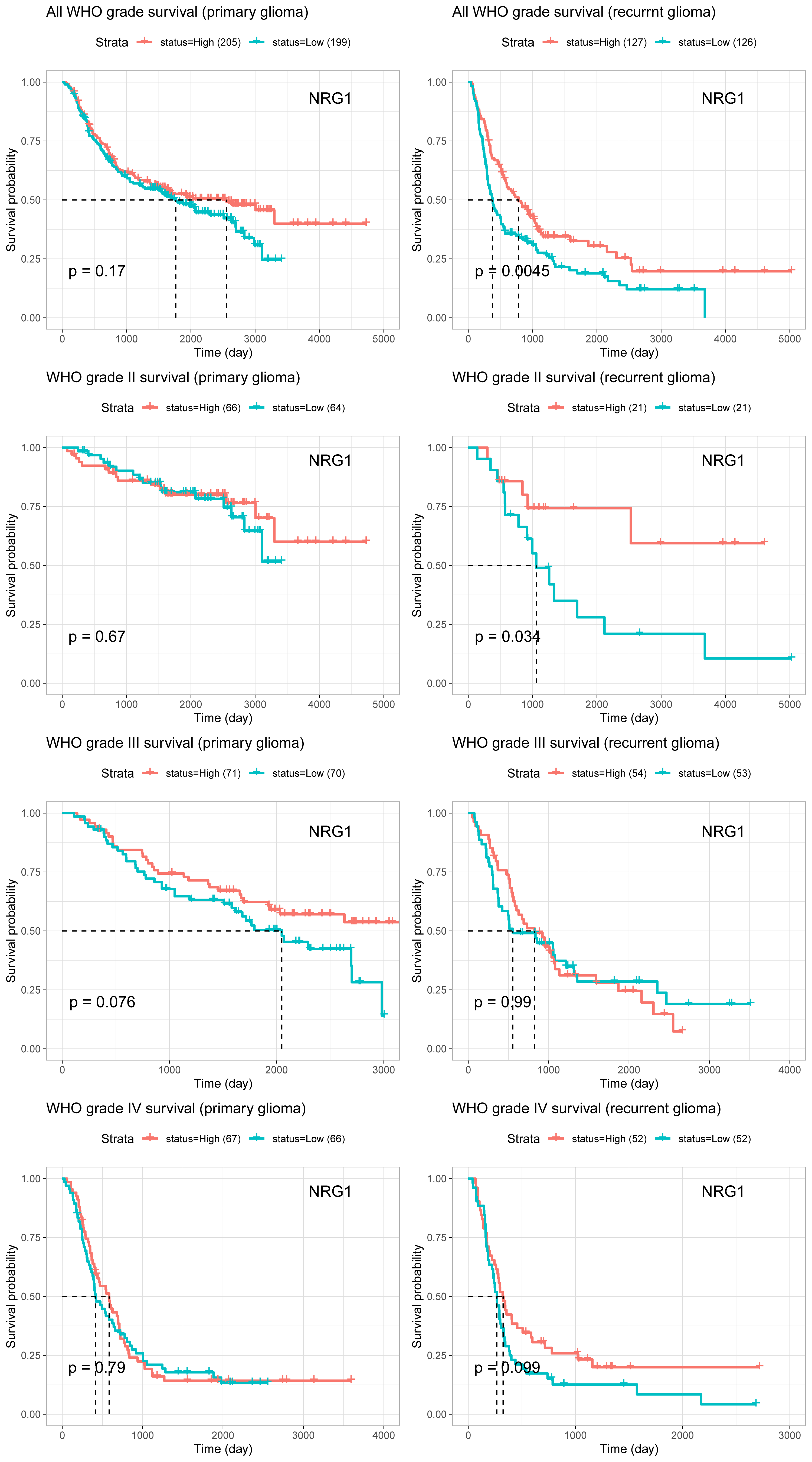

Supplement: Supplementary Figure 1 — The prognostic value of NRG1 in primary and recurrent gliomas in different WHO grades. [file Image_1.tif]

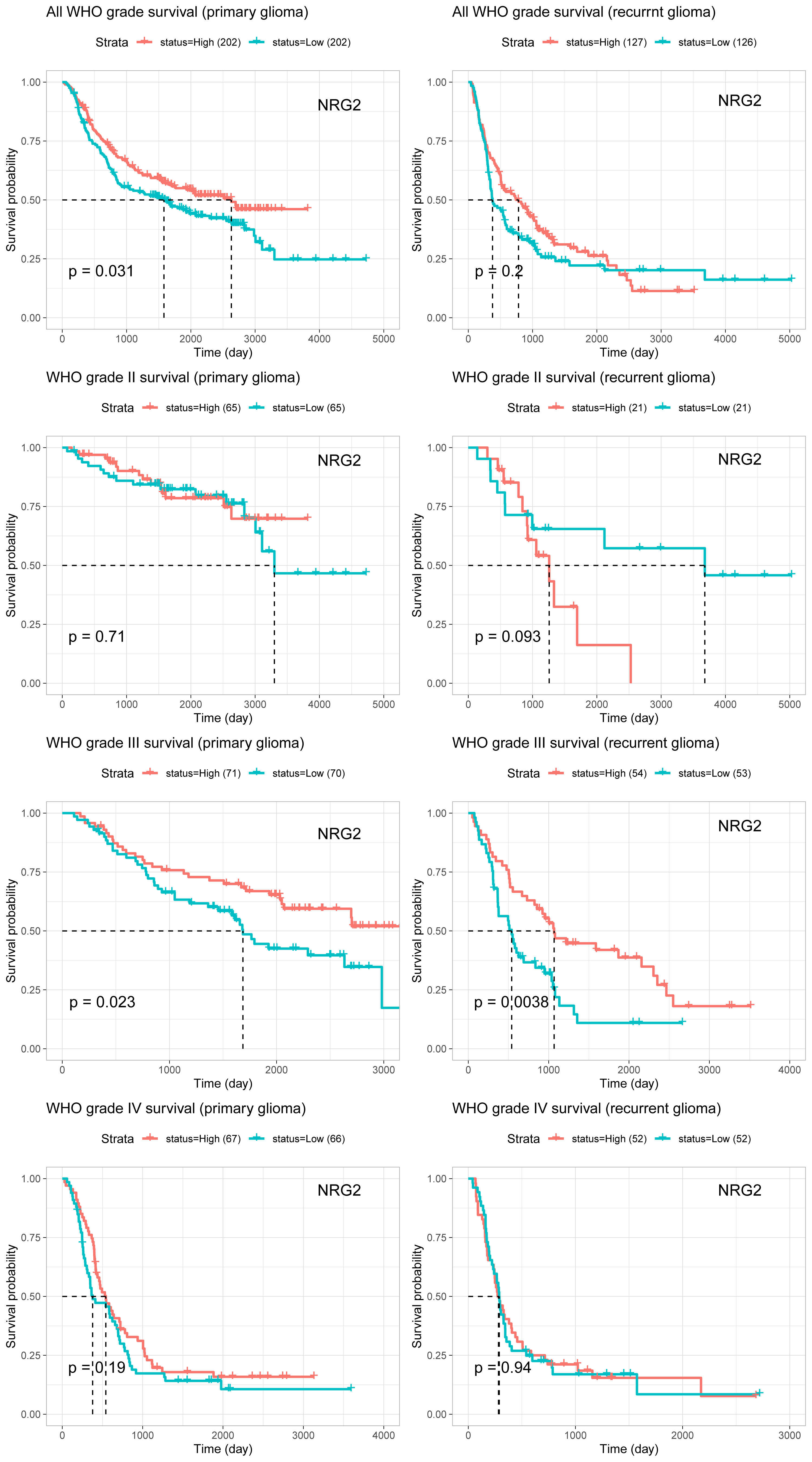

Supplement: Supplementary Figure 2 — The prognostic value of NRG2 in primary and recurrent gliomas in different WHO grades. [file Image_2.tif]

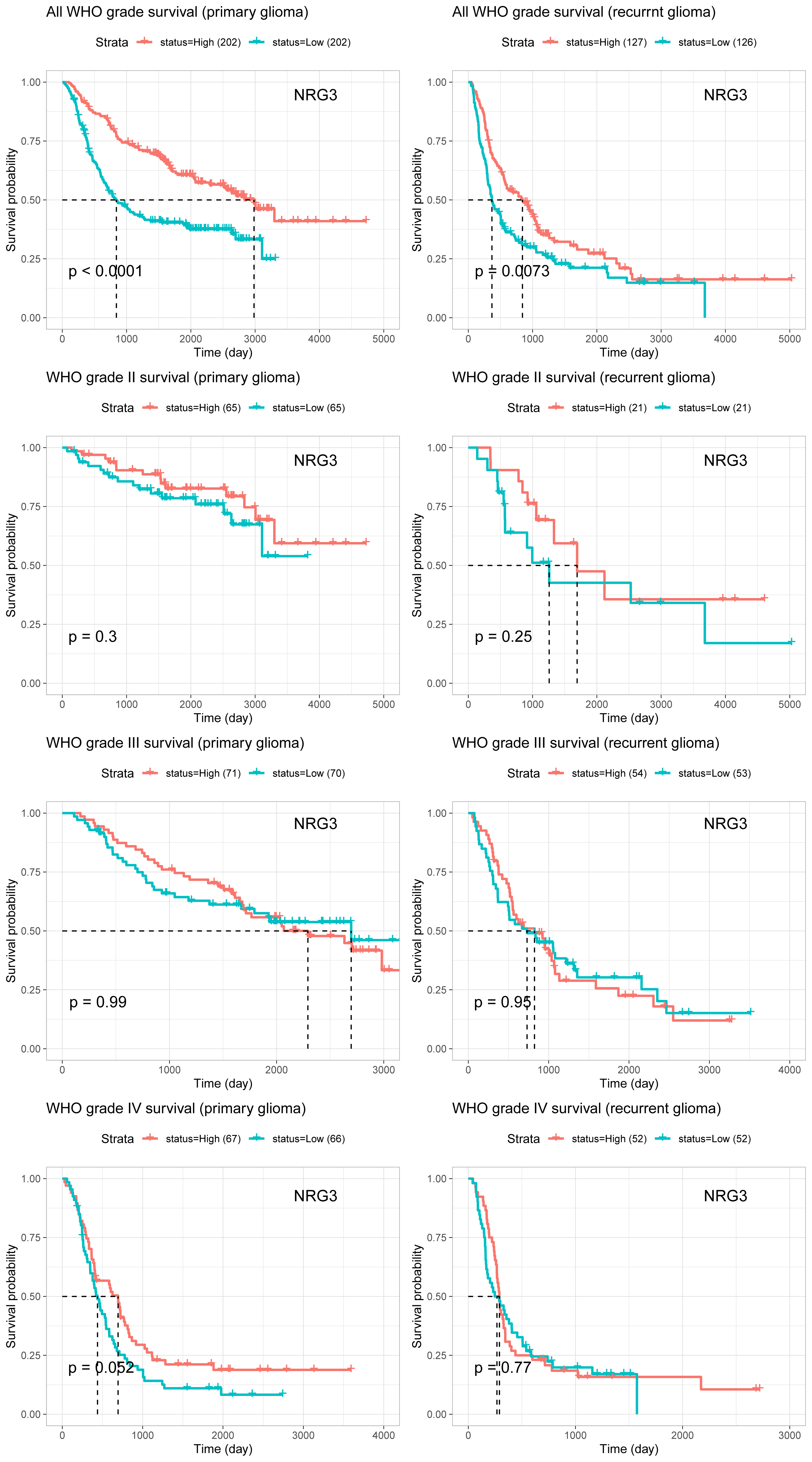

Supplement: Supplementary Figure 3 — The prognostic value of NRG3 in primary and recurrent gliomas in different WHO grades. [file Image_3.tif]

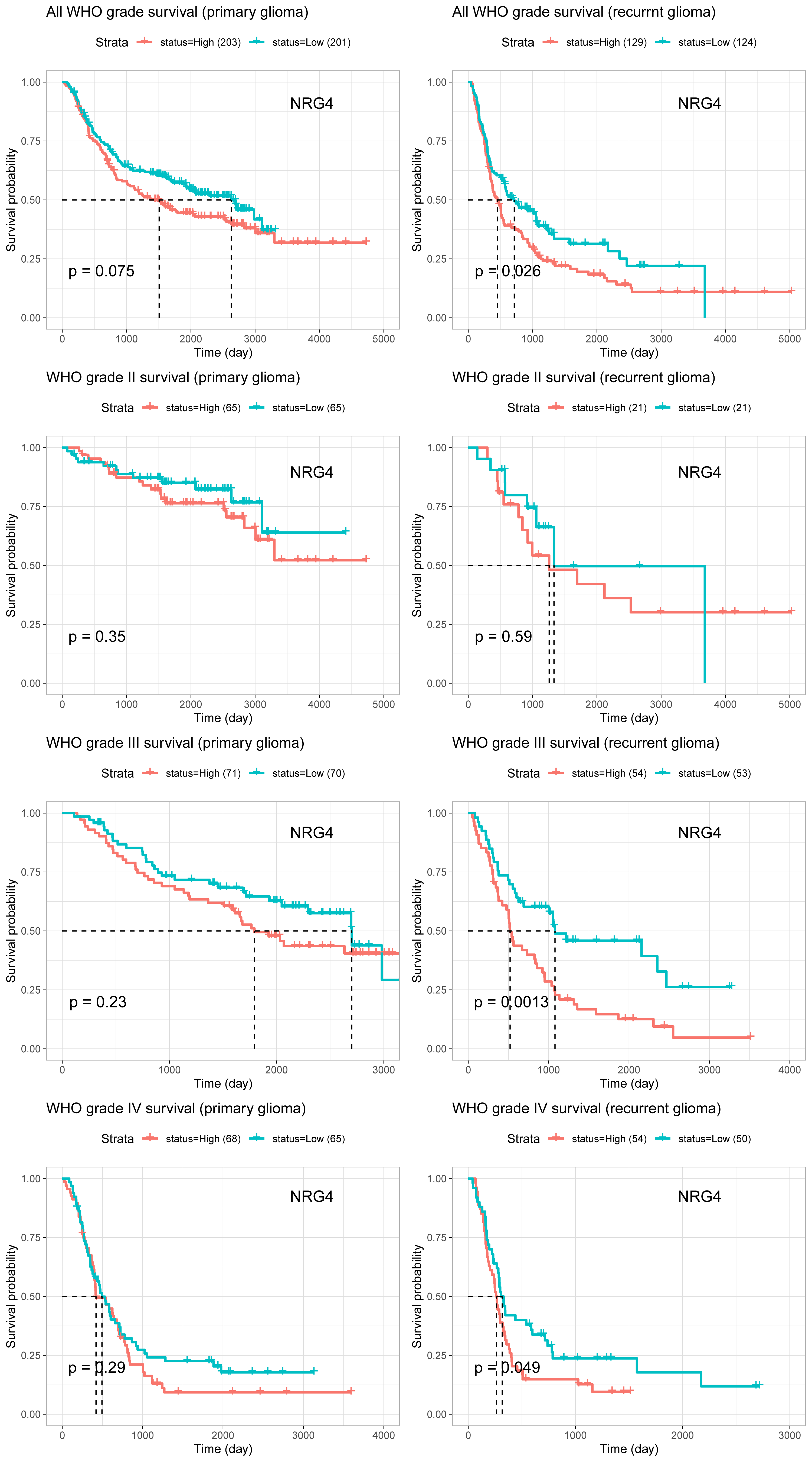

Supplement: Supplementary Figure 4 — The prognostic value of NRG4 in primary and recurrent gliomas in different WHO grades. [file Image_4.tif]
